# Se (IV) triggers faster Te (IV) reduction by soil isolates of heterotrophic aerobic bacteria: formation of extracellular SeTe nanospheres

**DOI:** 10.1186/s12934-014-0168-2

**Published:** 2014-11-26

**Authors:** Mini Bajaj, Josef Winter

**Affiliations:** Institute of Biology for Engineers and Biotechnology of Wastewater, Am Fasanengarten, Karlsruhe Institute of Technology, Karlsruhe, 76133 Germany

**Keywords:** Aerobes, Bioreduction, Selenite, Tellurite, Extracellular nanospheres

## Abstract

**Background:**

Selenium and Tellurium have many common chemical properties as both belong to group 16 of the periodic table. High toxicities of Se and Te oxyanions cause environmental problems in contaminated soils and waters. Three strains (C4, C6 and C7) of selenite reducing and nanoparticle forming aerobic bacteria which were isolated from agricultural soils of India containing high concentrations of Se were investigated after 3.5 months of freeze-storage for their resistance against the toxic oxyanion tellurite and its reduction to non toxic elemental form Te^0^ as well as nanoparticles formation.

**Results:**

Strains C4, C6 and C7 reduced tellurite at maximum reduction rates of 2.3, 1.5 and 2.1 mg Te (IV)/L/d, respectively and produced extracellular Te^0^ nanospheres as revealed from SEM-EDX analysis. Production of extracellular Te nanospheres has been described seldom. Further, concurrent reduction of both selenite and tellurite by bacteria was examined as these toxic oxyanions are often present together in natural environments, mine tailings or wastewater from copper refining. Interestingly, bioreduction of 100 mg/L selenite in shake flasks was not much affected by the presence of 10 mg/L tellurite but tellurite reduction rate increased 13 fold with selenite in the medium. The concurrent reduction of these oxyanions resulted in rarely described bioformation of extracellular nanoparticles composed of both Se and Te, reported first time for aerobically growing heterotrophic non-halophilic bacterial cultures. *Duganella violacienigra,* the closely related strain to C4 was also found to be resistant to oxyanions of Se and Te.

**Conclusions:**

Selenite reducing heterotrophic non-halophilic aerobic bacteria revived from 3.5 months freeze storage could successfully reduce toxic tellurite to non toxic elemental form and produced extracellular nanospheres during detoxification. Presence of relatively less toxic selenite in the medium triggers bioreduction of more toxic tellurite leading to formation of extracellular SeTe nanospheres which are sought by solar and optical recording media industry because of their excellent photovoltaic and optical properties. The bacterial cultures investigated in this study could be exploited commercially to remediate not only selenite and tellurite-contaminated soil and water but also for green synthesis of extracellular Se, Te and Se + Te nanospheres.

## Background

Selenium and tellurium share many common characteristics e.g. both metalloids are found with copper- and sulfur-bearing ores and exist in four oxidation states i.e. +VI, +IV, 0, and –II in nature. Oxyanions (IV and VI) of both elements are soluble and mobile thus bioavailable and toxic. Elemental (zero valent) Te and Se are non soluble. Both Te and Se are found in relatively low abundances in earth’s crust, yet high toxicities of these elements cause environmental problems in contaminated soils and waters e.g. in agricultural lands or wastewater discharges from industrial activities or mine tailings [1,2]. Due to an increased inclination towards biological treatments, microorganisms capable of reducing toxic oxyanions have been investigated by many researchers [3–10]. Relative to arsenic, not many studies discussing Se geo-chemical-microbial cycling in the environment have been reported and the studies focusing on Te are even less than Se. Till now, the biological function of Te and its microbial oxidation is not known. Since concentrations as low as 1 μg/L of tellurite are toxic to most bacteria [11,12], it is especially used in selection media for Te resistant microorganisms which are often pathogens, e.g. Shiga toxin-producing *E.coli* [9,13]. Owing to their resemblance to sulfur, Te and Se disrupt the thiol chemistry within cells leading to toxicity in bacteria not resistant to these compounds [9]. In the last decade, many anaerobic bacteria capable of both selenium and tellurium reduction to non toxic forms have been isolated from extreme environments like ocean hydrothermal vents [10,14] or the highly alkaline water of Monolake, California [7,8,15] but competent aerobic non-halophilic heterotrophic bacteria reducing both Se and Te are not yet known. Concurrent Te and Se reduction has also not been investigated for chalcogen reducing heterotrophic aerobic bacterial cultures except for a few studies describing synergistic reduction of Te + Se by halophilic bacteria [6,16,17].

As described previously [18], four strains of aerobic bacteria from Se enriched agricultural soils in India were isolated that were able to produce extracellular Se nanospheres during selenite reduction to Se^0^. The strains C1 and C4 (GenBank NCBI accession numbers JQ745646 and JQ745647) were found to be closely related to *Duganella violacienigra* species and strains C6 and C7 (GenBank NCBI accession numbers JQ745648 and JQ745649) were related to *Agrobacterium tumefaciens* species [18]. In the present study strains C4, C6 and C7 were revived after 3.5 months of freeze-storage and were investigated for their resistance against selenite as well as against tellurite. Tellurium is the next element to Se in chalcogen group 16 of the periodic table and shares many properties with each other. Scanning- and transmission electron microscopy (SEM and TEM) with energy dispersive X-ray (EDX) analysis were carried out to observe nanoparticle formation of elemental Se and Te produced as a result of detoxification. The effect of the presence of Se (IV) on Te (IV) reduction was further examined to elucidate bioremediation when multiple detrimental ions are present as it often occurs in natural environments or copper refining. Since isolate C4 was closely related to *Duganella violacienigra* [18] which is one of the few known species of this genus therefore, the type strain *D. violacienigra* DSM 15887 was also examined for its resistance against Te (IV) and Se (IV) as it has not been investigated before.

## Results and discussions

### Selenite reduction after revival of freezed cultures

**Figure 1:**
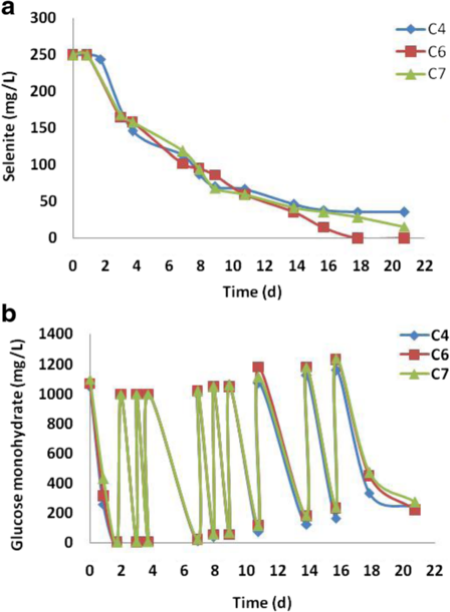
**Selenite reduction efficiency of bacteria.** Selenite reduction **(a)** and glucose degradation **(b)** by aerobic cultures of soil isolates C4, C6 and C7 after revival from freeze-storage.

Pure cultures of selenite-reducing aerobic bacteria that have been stored for 3.5 months in a deep freezer were revived by thawing at room temperature (25 ± 2°C) and incubation in media with glucose but without Se (IV). Initial growth of bacteria was slow but the growth rate accelerated during 3-4 successive incubations in fresh medium. After achieving a short lag phase, 5% inoculum from overnight cultures was inoculated in fresh medium with 1 g/L glucose monohydrate and 40 mg/L Se as 133.22 mg/L of Na_2_SeO_3_·5H_2_O (MW =263.01 g) for incubation at 27°C. The concentration of Se was increased during successive incubations up to 250 mg/L Se (IV) or 832.62 mg/L of Na_2_SeO_3_·5H_2_O in the medium. Selenite reduction was followed through ion chromatography showing gradual disappearance of Se (IV) over the days without appearance of any Se (VI) peaks indicating absence of Se (IV) oxidation. After 15.5 days of incubation 85% and 86% of 250 mg/L Se (IV) was reduced by C4 and C7, respectively, whereas 94% of Se (IV) was reduced by strain C6. Complete removal required less than 18 days (Figure 1a). Glucose in the medium was biodegraded within 2 days of incubation. More glucose was supplied in regular intervals to avoid carbon limitation during Se (IV) reduction (Figure 1b). Investigation of cultures with SEM-EDX revealed extracellular Se nanoparticles (figures not shown). Very few studies have been performed on the viability and cultivation of strains after storage of deep-freezed cultures. For revival, lengthy periods of incubation are often required, particularly for microbes that originate from oligotrophic habitats, where a non-growing or dormancy state may be the norm [19]. In their review, Joint et al. [20] also reported that transition from a ‘non-growing’ to a ‘growing’ state in a synthetic laboratory medium is critical and adaptation to growth conditions might be a very slow process. Bacterial cultures used in the present study were isolated from soil [1,18] and after freeze-storage, successive long incubations were necessary to revive the capability of the bacteria to form elemental nanoparticles by adapting them again to increasing concentration of Se (IV).

### Incubation with Tellurite

**Figure 2:**
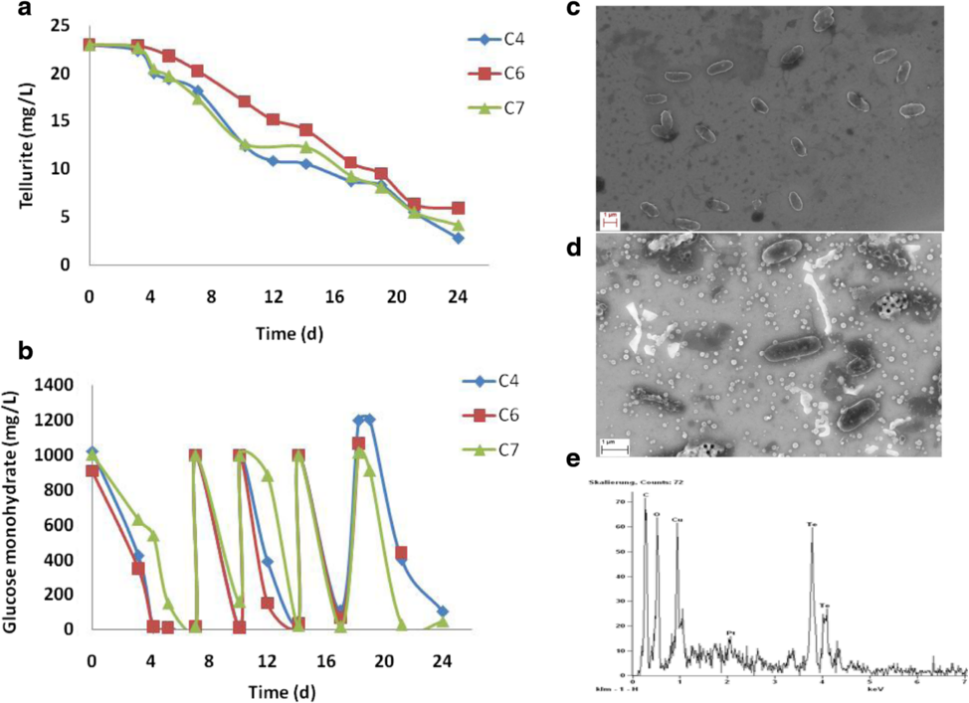
**Bioreduction of tellurite and nanoparticle formation.** Tellurite reduction **(a)** and glucose degradation **(b)** by aerobic cultures of soil isolates C4, C6 and C7. SEM microphotograph of culture C4 grown in the absence **(c)** and in the presence of Te (IV) **(d)**, showing Te nanoparticles, confirmed by SEM-EDX spectral analysis **(e)**.

Before conservation of cultures by freezing for 3.5 months, cultures had been incubated with tellurite. Within 2-3 days of incubation a change in color of the medium from colorless to black was observed (not shown). This visible observation was taken as an indication of reduction of colorless tellurite to its black colored elemental form Te (0). Since these cultures were able to retain their capacity to reduce selenite and to produce extracellular Se^0^ nanoparticles even after 3.5 months of freeze-storage (see above section), they were incubated with 45.75 mg/L of K_2_TeO_3_ (23 mg/L Te) to investigate their Te (IV) reduction capability after revival. In all assays the medium changed from colorless to black and darkened up towards the end of incubation. Average Te (IV) reduction observed in the assays of strains C4, C6 and C7 after 24 days of incubation was 88%, 74% and 82%, respectively, with maximum removal rates of 2.3, 1.5 and 2.1 mg Te (IV)/L/d (Figure 2a). In parallel, quick glucose degradation was observed in all assays requiring re-feeding of glucose at regular intervals (Figure 2b). It was assumed that if these strains could reduce Te (IV), then they might also form Te (0) nanoparticles in analogy to Se nanoparticle formation, mimicking the Se detoxification mechanism. Therefore, SEM analyses of these cultures along with respective controls were carried out. All three Strains C4, C6 and C7 could produce extracellular Te nanospheres, respectively which were confirmed with EDX analysis. The nanoparticles produced by e.g. strain C4 are shown in Figure 2c, d and e. The size of Te nanoparticles formed by the three strains ranged from ca. 33 to 220 nm with the majority of them having a diameter around 100 nm. As a p-type semiconductor tellurium nanomaterials have a wide range of applications in photovoltaic cells, light-emitting diodes, sensors and nano devices due to their good photoconductivity, thermoelectricity, catalysis, high piezoelectricity, nonlinear optical properties and ability to form functional materials with many chemicals [21,22]. Successful application of such semiconductors depends on their morphology, structure, size and surface functionality. Therefore, new routes for manufacturing various Te nanostructures e.g. nanotubes, nanowires and nanospheres are being searched for [21].

Bacterial reduction of tellurite and its deposition occur almost exclusively intracellular [9,12,23]. Bioformation of extracellular Te nanoparticles is not common and almost all reports describe extracellular deposits as nanorods or needle like nanoprecipitates [4,5,24]. The formation of extracellular Te nanospheres was only described by the Te (IV) respiring anaerobic *Sulfurospirillum barnesii* [7]*.* This strain also reduced Se (IV) and other metalloids as terminal electron acceptors [7,8]. Production of extracellular spherical Te nanoparticles by heterotrophic aerobic bacteria has not yet been reported. Only a few studies described extracellular Te deposition by aerobic bacteria as Te nano rods [25] or nanoparticles of up to 300 nm with an undefined shape [3]. Recently, Borghese et al. [26] described reduction of Te (IV) and Se (IV) oxyanions into extracellular needle like Te^0^ and globular Se^0^ nano precipitates during anaerobic growth of the facultative photosynthetic bacterium *Rhodobacter capsulatus*. The formation of extracellular nano precipitates was dependent on the carbon source that was supplied for bacterial growth. The rate of chalcogen reduction was constant at different concentrations of lawsone that was added as a redox mediator to facilitate the formation of external Te nanoparticles. In the present study, no external redox mediator was required but glucose as a carbon source was essential and effective for both Se (IV) and Te (IV) reduction leading to spherical nanoparticles of Se (0) and Te (0) [[18]; Figure 2d and e) which is reported first time for aerobically growing heterotrophic non-halophilic bacterial cultures. Extracellular nanoparticles could be separated from bulk media and might be used for possible applications where a high surface to volume ratio of spherical shape is advantageous.

### Incubation of cultures in the presence of both Selenite and Tellurite

**Figure 3:**
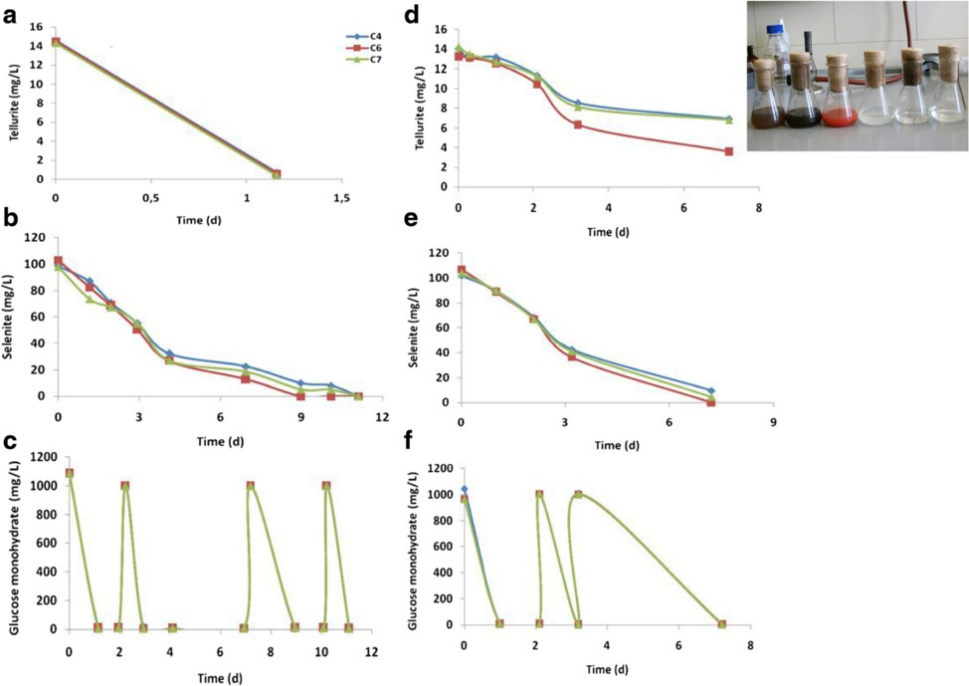
**Influence of selenite on tellurite reduction.** Tellurite reduction **(a)**, selenite reduction **(b)** and glucose degradation **(c)** when present concurrently. Reduction of Te (IV) in the absence of Se (IV) **(d)** and reduction of Se (IV) **(e)** during aerobic glucose degradation **(f)** in absence of Te (IV). Inset photo showing color change of medium (L to R) in first three flasks after tellurite + selenite-, tellurite- and selenite-reduction, respectively. Turbidity was observed in control (4^th^ flask) containing no chalcogens after growth of inoculum with glucose whereas the medium remained colorless in sterile controls (5^th^ and 6^th^ flask) containing only tellurite as well as tellurite + selenite, respectively.

**Figure 4:**
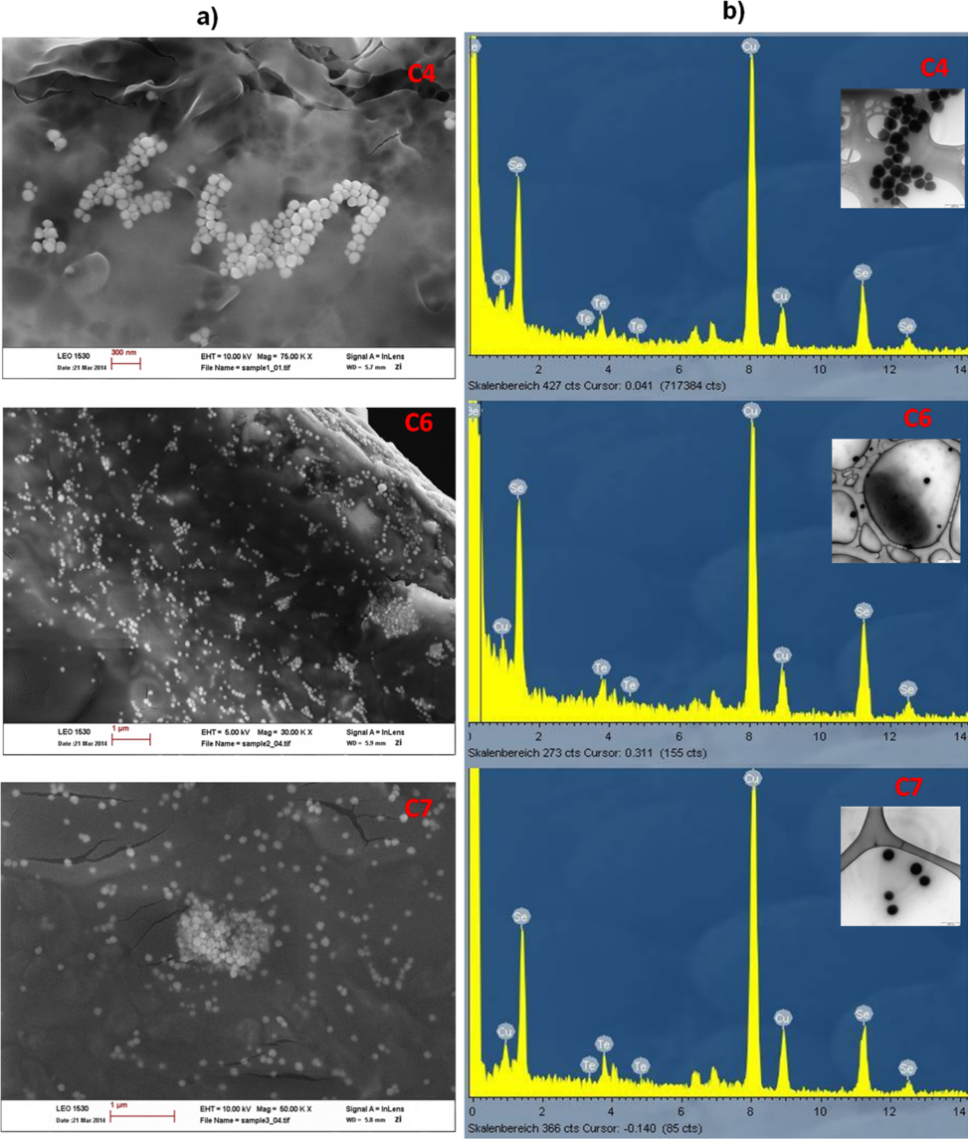
**Extracellular production of SeTe nanospheres.** SeTe nanospheres synthesized by cultures C4, C6 and C7 **(a)** along with respective TEM-EDX spectra **(b)**. Inset showing TEM microphotographs of nanospheres on carbon coated copper grids.

In the previous experiments (see above section) it was confirmed that isolates C4, C6 and C7 were capable of reducing tellurite which is approximately 100-fold more toxic to bacteria than selenite [9,27]. To investigate combined ion detoxification, strains C4, C6 and C7 were incubated in the presence of both Se and Te oxyanions. In the presence of 100 mg/L of Se (IV), the three strains reduced 14 mg/L of Te (IV) in 1.2 days (Figure 3a) and almost 100 mg/L Se (IV) in about 9 days (Figure 3b) during regular re-feeding of 1 g/L glucose after its degradation (Figure 3c). The control assay that contained only 14 mg/L of Te (IV) and no Se (IV) required a longer time for a non-complete reduction of tellurite (Figure 3d) indicating that Te (IV) reduction was stimulated by parallel reduction of a similar amount of Se (IV). Selenite reduction in the presence or absence of Te (IV) occurred at about the same rate (Figure 3b, e). Reduction of chalcogens was not observed in sterile controls which was also confirmed by lack of color change in the medium (Figure 3 inset). On the fourth day of incubation, samples were taken for SEM- and TEM-EDX analysis to investigate formation and composition of nanoparticles. When the samples were taken, 100% of 14 mg/L Te (IV) and 67.7%, 73.3% and 73.0% of 100 mg/L Se (IV) in the medium was reduced by the cultures of strains C4, C6 and C7, respectively. With SEM, spherical nanoparticles with a diameter ranging from 50 to 150 nm were observed in culture C4, 70 to 140 nm in culture C6 and 86 to 131 nm in culture C7. The majority of nanoparticles formed by cultures C4, C6 and C7 had a diameter of 100 nm (Figure 4a). SEM-EDX analyses indicated Te and Se to be the elemental constituents of these particles. Since SEM-EDX analyses covered an area of about 100 nm, more precise TEM-EDX analyses with a spot size of 12 nm were performed. Spectra of nanoparticles of the three cultures (Figure 4b) again confirmed that each particle consisted of both Se (0) and Te (0). The Cu and Be peaks in the spectra (Figure 4b) were due to the instrumental contamination caused by the copper grid used as specimen stage and the beryllium window in TEM-EDX. No other study except the one by Pearce et al. [5] reported bioformation of nanospheres composed of both semi-conductive metalloids, Se + Te. In the study of Pearce et al. [5] *B. beveridgei* strain MLTeJB reduced up to 10 mM Te (IV) to Te (0) as the sole reduction product under anaerobic conditions, when excess lactate was present. Subsequent addition of 5 mM of Se (IV) to the suspension after Te (IV) reduction to Te (0) resulted in reduction of Se (0) to Se (–II). SEM-EDX analysis revealed that cells grown first in the presence of Te (IV) and then with Se (IV) upon depletion of the former were encrusted with nanospheres composed of both Se and Te. The mechanism of such nanoparticle formation was not clear but it was assumed that the addition of Se (IV) to the Te (0) nanoparticles containing suspension stimulated cells of *B. beveridgei* strain MLTeJB to maintain a reducing environment in which further reduction of Te (0) to Te (–II) was possible. In the present study, Se (IV) was added concurrently with Te (IV) to the medium and both metalloids were at the same oxidation state at start. The advantage of Se + Te nanoparticles compared to elemental Se would be that amorphous SeTe nanostructures have greater hardness, higher crystallization temperature, higher photosensitivity and less ageing effects. As a consequence, they have several applications in optical recording media because of their excellent laser writer sensitivity, xerography, electrographic applications such as photoreceptors in photocopying and laser printing, infrared spectroscopy as well as switching and memory devices [28].

Se and Te oxyanions in the group of chalcogens can be reduced by both, anaerobic and aerobic bacteria. The majority of these reduction processes form chalcogen-containing organics e.g. volatile organometalloids and may produce elemental precipitates or nanoparticles through similar mechanisms via (1) enzymatic reduction, (2) methylation, (3) dissimilatory reduction concomitant with sulfate reduction, (4) Painter-type reactions with the thiols of proteins as well as glutathione and (5) a chemical reaction with the siderophore thiocarboxylic acid and the products of its hydrolysis [9,29]. Turner et al. [11] proposed a mechanism of tellurite toxicity in which tellurite oxyanions enter a cell via uptake by a phosphate transport system. Subsequently they were reduced to elemental tellurium by glutathione or other reduced thiols. Reactive oxygen species (O_2_^‐^) produced during reduction of tellurite may be detoxified by superoxide dismutase of aerobic bacteria. A similar mechanism was proposed by Kessi and Hanselmann [30] for selenite reduction in the glutathione synthesizing cyanobacteria and in α, β, and γ groups of proteobacteria (Gram-negative bacteria), thus being incompatible with strict anaerobes that lack oxidative stress enzymes. In the present study, all test strains were Gram negative proteobacteria. Strain C4 was phylogenetically closely related to β Proteobacteria, *Duganella violacienigra spec.* whereas, strains C6 and C7 were closely related to α Proteobacteria, *Agrobacterium tumefaciens spec.* [18]. The presence of Se oxyanions might have triggered glutathione reductase and other enzymes required to counteract Te (IV) and reactive oxygen species. A positive effect of Se oxyanions on tellurite reduction has previously only been reported by Amoozegar et al. [6] who observed a 25% increase in the removal of 0.5 mM tellurite from a 10% NaCl containing nutrient broth in the presence of 6 mM sodium selenite by a halophilic bacterium, *Salinicoccus* strain QW6, which was isolated from salty environments of Iran. In addition, in their recent study [16], improved reduction of 0.1 mM and 0.2 mM Te (IV), respectively by halophilic *Halomonas elongata* DSM2581 and halotolerant *Enterococcus faecalis* PTCC1237 was reported in the presence of Se (IV). Increased Te (IV) reduction efficiency in presence of Se (IV) particularly by halophilic or halotolerant bacteria was assumed to be due to the presence of sodium and potassium cations in tellurite and selenite solutions, which were necessary not only for growth and survival of the bacteria but also for enzymatic activities. Alternatively, it might be due to tellurite reductase induced by selenite ions [6,16,17]. However, in the present study of non-halophilic/halotolerent aerobic bacteria, Se (IV)-triggered Te (IV) reduction might be due to an increased reductase activity rather than due to an effect on Na^+^/K^+^ -ATPase influx.

### Tellurite reduction at various selenite concentrations and effect of tellurite re-feeding

**Figure 5:**
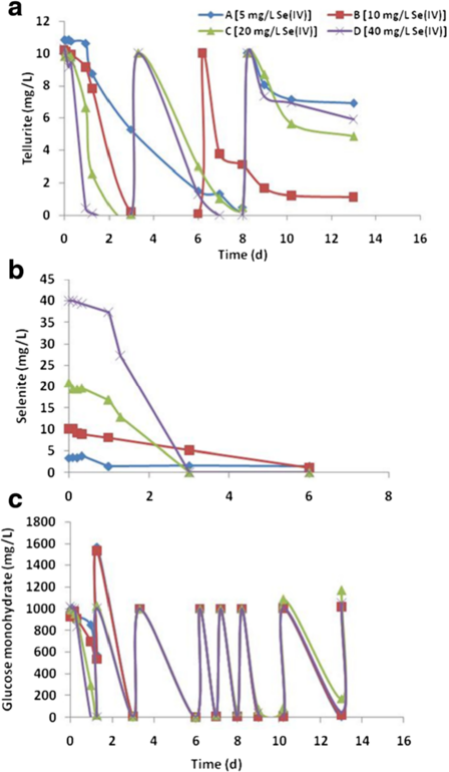
**Effect of different concentrations of Se (IV) on concurrent Te (IV) reduction.** Te (IV) reduction in presence of Se (IV) **(a)**, reduction of different concentrations of Se (IV) **(b)** along with glucose degradation **(c)** by strain C6.

No Se (IV) or Te (IV) reduction was observed in assays when both oxyanions were present simultaneously at concentrations of 100 mg/L each in the medium. However, in the presence of 10 mg/L Te (IV) and 100 mg/L Se (IV) the maximum reduction rate of tellurite was 29.41 mg Te (IV)/L^.^d (data not shown). Reduction of Te (IV) was greatly influenced by the presence of excess Se (IV). To investigate the effect of the concentration of Se (IV), 10 mg/L of Te (IV) was incubated with 5 to 40 mg/L of Se (IV) representing ratios of 2:1, 1:1, 1:2 and 1:4 Te (IV):Se (IV), respectively in the medium. Except for controls, media were inoculated with test strain C6. It was observed that Te (IV) reduction was fastest in the assays with the highest Se (IV) concentration, and Te (IV) reduction rates decreased in the order of decreasing Se (IV) concentrations (Figure 5a). It took just one day for removal of 10 mg/L Te (IV) when Se (IV) was present in a ratio of 1:4 whereas in a parallel assay 8 days were required for reduction of a similar amount of Te (IV) when the Te (IV):Se (IV) ratio was 2:1. No significant change in concentrations of both Se and Te (measured at start and at the end of experiments) or the change in media color was observed in sterile controls omitting the possibility of chalcogen reduction via abiotic processes. It was obvious that Se (IV) oxyanions induced detoxification of Te (IV) resulting in faster reduction of tellurite. When the concentration of selenite was low, a quick reduction of Te (IV) for detoxification was not observed, resulting in partial inhibition of growth of the bacteria due to toxicity of Te (IV) anions in the medium. Apparently, Se (IV) reduction rates were much slower in assay with low Se (IV) concentrations than in the assays with much higher concentrations of Se (IV) (Figure 5b). After complete reduction of Te (IV) and Se (IV) in assays C and D (Figure 5a and b), 10 mg/L of Te (IV) was fed again in both assays. Even after complete Se (IV) reduction in these assays, the Te (IV) reduction rates at successive re-feed were much faster compared to assays incubated with Te (IV) only (Figure 3d). During this period glucose was re-fed twice after complete reduction of the second feeding of 10 mg/L of Te (IV) in assay D and in assay C (Figure 5a and c). At the time of the third feeding of 10 mg/L of Te (IV) in assay C and D (Figure 5a), the bacteria were growing already without both oxyanions for a while and apparently have inactivated detoxification enzymes resulting in slower tellurite reduction comparing to first two feedings.

### Tellurite and Selenite reduction by *Duganella violacienigra*

**Figure 6:**
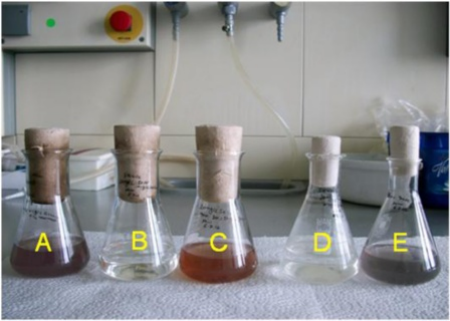
**Selenite and tellurite reduction by** ***Duganella violacienigra.*** **A)** Typical violet pigments are visible when *D. violacienigra* was grown without Te (IV) or Se (IV) in the medium, **B)** Absence of color in sterile medium containing Se (IV), **C)** Red color in medium with Se (IV) indicating Se (IV) reduction, **D)** Absence of color in sterile medium containing Te (IV) and **E)** Black color indicating Te (IV) reduction by *D.violacienigra.*

In our previous study, strain C4 was identified to be 95% phylogenetically similar to *Duganella violacienigra* [18]. *Duganella* spec. are members of β-Proteobacteria within the family Oxalobacteraceae, comprising Gram-negative strictly aerobic/anaerobic bacteria. Only a few bacteria are classified within this genus [31] and their tolerance to toxic oxyanions of selenite or tellurite is not yet known. To investigate selenite and tellurite resistance, *D. violacienigra* DSM 15887 was grown in the absence and in the presence of 20 mg/L of Se (IV) and Te (IV), respectively. After 3 days of incubation a color change from colorless to violet was observed in control assays without Se (IV) or Te (IV) and to red or black in the medium containing Se (IV) or Te (IV), respectively. This indicated oxyanions reduction to their respective elemental forms (Figure 6). *Duganella* species isolated from agricultural soils [18,31] and presumably other soil bacteria thus might be playing a significant role in Se and Te cycling in soil and contribute to oxyanion detoxification by reducing selenite or tellurite even under aerobic growth conditions. These microorganisms could be exploited commercially for bioreduction of toxic oxyanions of Te and Se to remediate selenite and/or tellurite-contaminated soil and water.

## Materials and methods

### Microbial cultures

Isolation and identification of bacteria C4, C6 and C7 and their main physicochemical properties have been described in detail previously [18]. All bacteria were isolated from mixed microbial aerobic cultures enriched from a high selenium containing soil obtained from agricultural fields in India. After log-phase growth of the pure cultures in medium without Se (IV) (according to Ghosh et al. [32]), the cell suspension was mixed with 86% w/v glycerol in the ratio of 3:1 and stored at -20°C in a deep freezer. After 3.5 months of storage the cultures were thawed and 2 ml of respective cultures were suspended in 20 ml medium containing 1 g/L glucose monohydrate and no Se (IV) in 100 ml shake flasks with cotton plugs. Flasks were incubated at 27°C on a rotary shaker at 110 rpm. These bacteria were adapted to high Se (IV) concentrations in successive incubations in fresh medium (40 ml) containing 1 g/L glucose monohydrate and increasing concentrations of Se (IV) with 30-40 mg/L increments during 1 to 1.5 months.

### Experimental design

#### Chalcogens resistance assays

Growth medium [32] containing 0.03 g NH_4_Cl, 0.01 g NaCl, 0.01 g MgSO_4_, 0.15 g yeast extract and 0.5 g peptone per L of deionized water, pH 7.5 supplemented with 1 g/L glucose monohydrate and the indicated concentrations of Se (IV) as Na_2_SeO_3_·5H_2_O or Te (IV) as K_2_TeO_3_ (Sigma-Aldrich, Taufkirchen or Serva, Heidelberg, Germany) in 100 ml Erlenmeyer flasks was inoculated with 5% of pure cultures of the bacterial strains C4, C6 and C7, respectively. Concentrated solutions of each C_6_H_12_O_6_·H_2_O, Na_2_SeO_3_·5H_2_O and K_2_TeO_3_, respectively were sterilized separately and added to growth medium under sterile conditions as required. Controls containing inoculum and no chalcogens as well as sterile controls containing Se (IV), Te (IV) or both Se (IV) and Te (IV), respectively were carried out for all assays. Flasks were closed with cotton plugs and incubated at 27 ± 2°C on a rotary shaker at 110 rpm. Working volumes of the assays were 40 to 60 ml and the bacterial concentration was about 1.8 to 2.2 ×10^7^ cells/ml in the flasks at the start of incubation.

#### Growth of *Duganella violacienigra* with Te and Se

A culture of *D. violacienigra* (DSM 15887) also known as *Pseudoduganella violaceinigra* [33] was purchased from Deutsche Sammlung für Mikroorganismen und Zellkulturen (DSMZ), Braunschweig, Germany. A loop full of preserved bacteria was inoculated in 100 ml Erlenmeyer flasks containing 50 ml medium [32] with 0.5 g/L glucose monohydrate and incubated at 30°C on a shaker at 110 rpm. After appearance of the violet pigmentation, 10% of inoculum from this culture was transferred into fresh medium containing 20 mg/L of Se (IV) or Te (IV), respectively and 1 g/L glucose monohydrate. Conditions for incubation were same as described above. Control assays with only bacteria but without Te or Se and two sterile assays containing Se (IV) and Te (IV), respectively, in the medium were run in parallel.

#### Analyses

Selenate and selenite were determined by ion-exchange-chromatography (ICS 90, Dionex) as described earlier [18]. Tellurite in the samples was measured according to Turner et al. [34] by binding of Diethyldithiocarbamate (DDTC) with Te (IV) in the presence of Tris-HCl buffer and analyzing absorbance at 340 nm with a spectrophotometer. Glucose was determined with dinitrosalicylic acid-reagent according to Miller [35] by measuring the absorbance with a spectrophotometer at 550 nm. Nanoparticle formation was observed and characterized by electron microscopy equipped with energy dispersive spectroscopy as described previously [18]. All chemicals used for analyses and medium were purchased from Carl-Roth, Karlsruhe, Germany except where stated otherwise.

## Conclusions

After 3.5 months of freeze-storage, bacterial cultures C4, C6 and C7 isolated from soil, were revived and could retain their selenite reduction activities for soil detoxification. Beside selenite they also reduced tellurite and formed extracellular Te (0) nanospheres, which is a rare phenomenon during aerobic growth. Not much is known about detoxification of selenite and tellurite in aerobic conditions, while more information exists on the anaerobic respiration of these oxyanions. It was also possible to reduce both oxyanions concurrently and the presence of selenite induced much faster tellurite reduction rates. Owing to their similar structure and chemical properties, selenite might have triggered the uptake and reduction of more toxic tellurite during detoxification mechanism by bacteria. The optimum ratio of selenite : tellurite for faster tellurite reduction was 4:1. The three soil isolates could also produce rarely described extracellular nanospheres composed of both, Se + Te. *Duganella violacienigra* that is phylogenetically very close to strain C4, could also reduce both oxyanions. The capability of reducing selenite and tellurite particularly in aerobic conditions makes these strain worthy of possible exploitation in bioremediation as well as for environment friendly production of useful nanoparticles.
